# Exposure to ambient air pollutions and its association with adverse birth outcomes: a systematic review and meta-analysis of epidemiological studies

**DOI:** 10.3389/fpubh.2024.1488028

**Published:** 2024-11-13

**Authors:** Belay Desye, Gete Berihun, Abebe Kassa Geto, Leykun Berhanu, Chala Daba

**Affiliations:** ^1^Department of Environmental Health, College of Medicine and Health Sciences, Wollo University, Dessie, Ethiopia; ^2^Department of Environmental Health, College of Medicine and Health Sciences, Debre Markos University, Debre Markos, Ethiopia; ^3^Department of Nursing and Midwifery, Dessie Health Science College, Dessie, Ethiopia

**Keywords:** ambient air pollution, outdoor air pollution, adverse birth outcomes, preterm birth, low birth weights, stillbirth

## Abstract

**Introduction:**

Air pollution is a significant global public health concern. However, there is a lack of updated and comprehensive evidence regarding the association between exposure to ambient air pollution and adverse birth outcomes (preterm birth, low birth weight, and stillbirth). Furthermore, the existing evidence is highly inconsistent. Therefore, this study aims to estimate the overall association between ambient air pollution and adverse birth outcomes.

**Methods:**

In this study, initially a total of 79,356 articles were identified. Finally, a total of 49 articles were included. We conducted compressive literature searches using various databases, including PubMed, Scientific Direct, *HINARI*, and Google Scholar. Data extraction was performed using Microsoft Excel, and the data were exported to STATA 17 software for analysis. We used the Joanna Briggs Institute’s quality appraisal tool to ensure the quality of the included studies. A random effects model was employed to estimate the pooled prevalence. Publication bias was assessed using funnel plots and Egger’s regression test.

**Results:**

In this study, the pooled prevalence of at least one adverse birth outcome was 7.69% (95% CI: 6.70–8.69), with high heterogeneity (*I*^2^ = 100%, *p-value* < 0.001). In this meta-analysis, high pooled prevalence was found in preterm birth (6.36%), followed by low birth weights (5.07%) and stillbirth (0.61%). Exposure to PM_2.5_ (≤10 μg/m^3^) throughout the entire pregnancy, PM_2.5_ (≤10 μg/m^3^) in the first trimester, PM_10_ (>10 μg/m^3^) during the entire pregnancy, and O_3_ (≤10 μg/m^3^) during the entire pregnancy increased the risk of preterm birth by 4% (OR = 1.04, 95% CI: 1.03–1.05), 5% (OR = 1.05, 95% CI: 1.01–1.09), 49% (OR = 1.49, 95% CI: 1.41–1.56), and 5% (OR = 1.05, 95% CI: 1.04–1.07), respectively. For low birth weight, exposure to PM_2.5_ (≤10 μg/m^3^) and PM_2.5_ (>10 μg/m^3^) throughout the entire pregnancy was associated with an increased risk of 13% (OR = 1.13, 95% CI: 1.05–1.21) and 28% (OR = 1.28, 95% CI: 1.23–1.33), respectively.

**Conclusion:**

This study highlighted a significant association between ambient air pollution and adverse birth outcomes. Therefore, it is crucial to implement a compressive public health intervention.

**Systematic review registration:**

The review protocol was registered with the record ID of CRD42024578630.

## 1 Introduction

Air pollution is a major global public health concern, with growing evidence linking exposure during pregnancy to a range of adverse birth outcomes (1, 2). Exposure to ambient air pollutants such as particulate matter (PM), ozone (O_3_), nitrogen dioxides (NO_2_), and sulfur dioxide (SO_2_) has been linked to a range of adverse birth outcomes, including preterm birth, stillbirth, low birth weight, and congenital anomalies (1, 3, 4). There is no evidence for the biological mechanisms underlying these associations, but they are thought to involve disruption of placental function, inflammation, and oxidative stress (5, 6). The World Health Organization (WHO) estimates that ambient air pollution caused 4.2 million premature deaths globally in 2019, highlighting significant implications for maternal and child health (7).

The effects of ambient air pollution on pregnancy can be attributed to both direct biological mechanisms and indirect socio-environmental factors. For instance, exposure to high levels of PM during critical periods of fetal development can disrupt placental function and fetal growth (8). Socioeconomic disparities often exacerbate the risks associated with air pollution, as marginalized communities frequently reside in areas with higher pollution levels and limited access to healthcare resources (9). Furthermore, the adverse effects of ambient air pollution on fetal development may have long-term consequences, as preterm birth and low birth weight are risk factors for various health problems later in life, including neurological disorders, cardiovascular disease, and diabetes (10).

A review with meta-analysis has revealed that prenatal exposure to ambient PM_2.5_ is associated with an increased risk of stillbirth (11, 12) and decreased birth weights (13). According to Lamichhane et al. (3) and Sun et al. (13), a 10 μg/m^3^ increase in PM_2.5_ exposure during pregnancy was associated with a 15 and 13% higher risk of preterm birth, respectively. Zhu et al. (4) reported that a 10 μg/m^3^ increase in PM_2.5_ exposure during pregnancy was associated with a 5% increased risk of low birth weight and a 10% increased risk of preterm birth. Similarly, Stieb et al. (1) concluded that there is consistent evidence linking air pollution exposure to preterm birth and low birth weight. Maternal exposure to PM_2.5_ (per 10 μg/m^3^ increased) was associated with a 15% increased risk of stillbirth in the entire pregnancy and a 9% increased risk of stillbirth in the third trimester (12). Exposure to major air pollutants throughout pregnancy may increase the risk of low birth weight (14). Several other studies have also indicated possible associations between ambient air pollution and adverse birth outcomes (15–20).

Although several reviews and meta-analyses have explored the association between specific ambient air pollutants and adverse outcomes, their findings have been inconsistent and lack comprehensiveness. Additionally, the conflicting results from previous primary studies underscore the need for a more thorough and integrated analysis of the available evidence. The purpose of this research is to thoroughly estimate the pooled association between ambient air pollutants and adverse birth outcomes, including preterm birth, low birth weight, and stillbirth, while also identifying predictive factors. Up-to-date and comprehensive evidence is crucial for informed decision-making, the development of effective strategies, and support for policymakers and other stakeholders.

## 2 Methods

**Figure 1:**
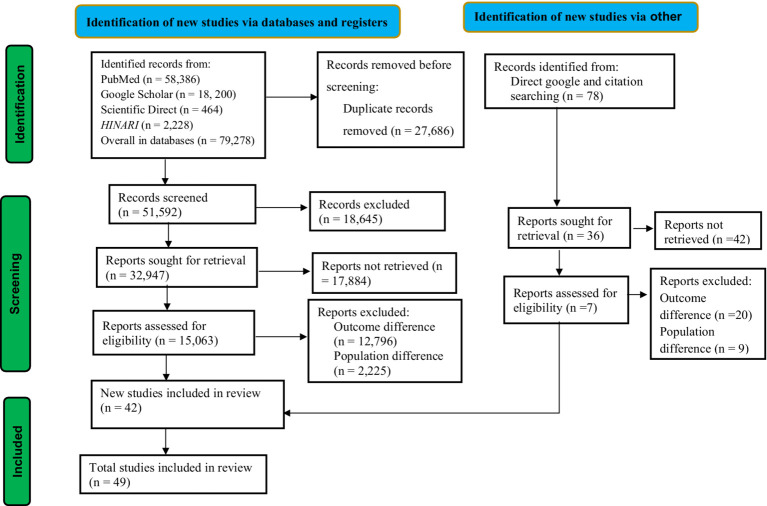
PRISMA flow diagram of association between ambient air pollution and adverse birth outcomes, 2024.

This study followed the guidelines of the Preferred Reporting Items for Systematic Reviews and Meta-Analysis (PRISMA) (21) (Figure 1). The review protocol for this study was registered in the International Prospective Register of Systematic Reviews (PROSPERO) with the record ID of CRD42024578630.

### 2.1 Eligibility criteria

#### 2.1.1 Inclusion criteria

The eligible for this review must meet the following PECOS (Participants/Populations, Exposures, Comparators, Outcomes and Study designs) criteria.

- Participants or populations: the participants are pregnant women at any stage of pregnancy up to birth.
- Exposures: prenatal exposure to ambient air pollution.
- Comparators: pregnant women with lower exposure levels, with or without adverse birth outcomes, as compared to those exposed to higher exposure with adverse birth outcomes.
- Outcomes: the adverse birth outcomes of interest include preterm birth, low birth weight, and stillbirth (reported prevalence [%] and measure of association in adjusted odds ratio [AOR]).
- **Study design**: all observational studies (cross-sectional, cohort, and case control).

Moreover, articles written in English, both published and unpublished, and studies reported from January 1, 2015 to August 30, 2024 were also the inclusion criteria.

#### 2.1.2 Exclusion criteria

Studies that investigated other pregnancy related outcomes besides the specified adverse birth outcomes (preterm birth, low birth weight, and stillbirth). Descriptive epidemiological studies (e.g., descriptive cross-sectional, case reports, and case series), studies without a full report after three personal email contacts with the primary and corresponding authors, conference abstracts, letters to the editors, qualitative studies, systematic reviews, short communications, and commentaries were not considered.

#### 2.1.3 Operational definitions

**Low birth weight**: “a birth weight of <2,500 g” (22).

**Stillbirth**: “A baby who dies after 28 weeks of pregnancy, but before or during birth” (23).

**Preterm birth**: “babies born alive before 37 weeks of pregnancy” (24).

In this study, we categorized the reported concentrations of pollutants into two main groups: ≤10 μg/m^3^ and >10 μg/m^3^. This categorization was necessary due to the variability in concentration levels reported across different studies.

### 2.2 Information sources

Both published and grey literature were sources of information for this study. A systematic literature search was undertaken using the following databases: PubMed, Scientific Direct, Google Scholar, and HINAR. The search was conducted for studies published from January 1, 2015, to August 30, 2024. In addition to the electronic database search, further articles were obtained by searching for grey literature through direct Google searches and by reviewing the references of the eligible studies.

### 2.3 Search strategies

Comprehensive search terms were used to identify relevant studies. These include MeSH terms and key words such as ambient air pollution, outdoor air pollution, adverse birth outcomes, preterm birth, low birth weights, and stillbirth. These search terms were used within PubMed as a template database to finalize an advanced search strategy utilizing the Boolean operators “AND” and “OR.” The search strategy was modified as appropriate for other databases and other sources.

### 2.4 Study screening and selection

All stages of reviewing articles were conducted independently by the two researchers (BD and AKG), with conflict managed by evidence-based discussion with the involvement of the third researcher (GB). From the search, the titles of all identified citations with abstracts were uploaded into Zotero reference manager, and duplicates were removed. Then it was followed by screening the titles and abstracts according to the eligibility criteria. The potential full text of eligible studies was retrieved. All studies that do not meet the inclusion criteria were excluded with reasons and presented in the PRISMA flow chart (21).

### 2.5 Quality (risk of bias) assessment of the selected studies

The quality of selected eligible studies was evaluated using the Joana Briggs Institute (JBI) critical appraisal checklist for cohort and case–control studies (25). The quality assessment was conducted independently by two reviewers (BD and ABK). In case of any discrepancies encountered during the quality assessment, they were managed through evidence-based discussions with the involvement of a third researcher (GB). Only studies that scored more than 50% on the quality assessment were considered for inclusion in this review (25, 26), as depicted in Table 1.

**Table 1:** Summary of the included studies on the association between ambient air pollution and adverse birth outcomes, 2024.

References	Country/region	Type of study	Exposure assessment	Pollutants	Outcome	Statistical Methods	Study period	Sample size	Cases	Prevalence (%)	Quality score (%)
Yang et al. (42)	China	Cohort	Daily mean concentrations of air pollutants	PM_2.5_, PM_10_, SO_2_, NO_2,_ O3, CO	SB	Logistic regressions	2011–2013	95,354	859	0.9	87.5
Quraishi et al. (43)	United States	Cohort	Regulatory and research monitors	PM_2.5_	PTB, LBW	Linear and Poisson regression	2006–2012	2,099	323	15.4	87.5
Chen et al. (44)	China	Cohort	Tracking Air Pollution	O_3_	PTB, LBW	Cox proportional-hazards regression model	2014–2016	56,905	4,835	8.5	87.5
Chen et al. (45)	Australia	Birth records	Daily air quality and meteorological data	PM_2.5_, SO_2_, NO_2_, O_3_	PTB, LBW	Cox-proportional hazards	2003–2013	173,720	24,702	14.2	87.5
Qian et al. (46)	China	Cohort	Daily measurement	PM_2.5_, CO, PM_10_, SO_2_, O_3_	PTB	Logistic regressions	2011–2013	95,911	4,308	4.5	75
Zhou et al. (47)	China	Cohort	National urban air quality monitoring	PM_2.5,_ PM_10_, O_3,_ CO, NO_2_, SO_2_	LBW	Generalized additive model	2015–2020	572,106	24,497	4.28	75
Chen et al. (48)	China	Birth records	Daily measurement	PM_10_, PM_2.5_, NO_2_, SO_2_	PTB	Cox proportional hazards regression models	2015–2017	13,111	614	4.7	87.5
Zhou et al. (49)	China	Cohort	Monitoring stations	PM_2.5_, PM_10_, SO_2_, CO, NO_2_, O_3_	PTB	Generalized additive model	2015–2020	572,116	33,669	5.88	87.5
Melody et al. (50)	Australia	Cohort	Annual estimation	PM_2.5,_ NO_2_	PTB, LBW	Linear and log-binomial regression models	2012–2015	285,594	23, 187	8.1	75
Li et al. (51)	China	Birth records	Daily measurement	PM_2.5_	PTB	Multilevel logistic models	2014	429,865	12,810	2.98	62.5
Zhao et al. (52)	China	Case–control	Monitoring stations	PM_10_	PTB	Logistic regression modeling	2010–2012	8,969	677	7.5	75
Padula et al. (53)	United States	Birth records	Daily measurement	CO, NO_2_, PM_10_, PM_2.5_	PTB	Logistic regression models	2000–2006	252,205	28,788	11.4	62.5
Tapia et al. (17)	Peru	Birth records	Ground measurements, satellite data, and a chemical transport model.	PM_2.5_	PTB, LBW	Linear and logistic regression model	2012–2016	123,034	10, 971	8.9	62.5
Liu et al. (54)	China	time-series	Ensemble-based models	PM_2.5_, PM_10_, SO_2_, NO_2_, O_3_, CO	PTB	General Additive model extend Poisson regression	2014–2016	37,389	5,428	14.5	87.5
Lavigne et al. (55)	Canada	Cohort	6 Digit-postal code captured	PM_2.5_, NO_2_, O_3_	PTB, LBW	Multivariable mixed-effect logistic regression	2005–2012	818,400	90,884	11.1	87.5
Huang et al. (56)	China	Birth records	Air monitoring data	PM_10_, NO_2_	PTB	Multi-pollutant models	2006–2010	50,874	3,203	6.3	87.5
Liu et al. (57)	China	Case–control	National environmental monitoring	PM_2.5_, PM_10_, SO_2_, NO_2_, CO, O_3_	PTB, LBW	Logistic regression models	2014–2015	86,139	1,784	2.1	87.5
Yorifuji et al. (58)	Japan	Cohort	Monitoring stations	SO_2_, NO_2_	LBW	multilevel logistic regression	200–2001	44,109	2,219	5	87.5
Stieb et al. (59)	Canada	Birth records	Ground-based monitoring data, estimates from remote-sensing, land use variables and, deterministic gradients relative to road traffic	PM2.5, NO2	PTB, LBW	Generalized estimating equations	1999–2008	3,104,090	242,150	7.8	75
Green et al. (60)	United States	Cohort	Air resources board	PM_2.5_, SO_2_, NO_2_, CO, O_3_	SB	Logistic regression models	1999–2009	5,788,117	26,355	0.5	75
Mendola et al. (29)	United States	Cohort	Community multiscale air quality	O_3_	SB	Poisson regression models	2002–2008	223,375	992	0.44	87.5
Ji et al. (61)	China	Case–control	Land use regression	NO_2_	PTB	Logistic regression	2014–2015	25,493	738	2.9	87.5
Guo et al. (62)	China	Cohort	National environmental monitoring	PM_2.5_	PTB	Cox proportional hazards regression	2014	426,246	35,261	8.3	75
Kingsley et al. (63)	United States	Birth records	Hybrid of land-use regression and satellite remote sensing	PM_2.5_	PTB	Linear and logistic regression models	2001–2012	61, 640	5,007	8.1	87.5
Ho et al. (64)	Vietnam	Time-series	Fixed monitoring stations	PM_2.5_	PTB, LBW	Linear and logistic regression model	2016–2019	163,868	18, 219	11.1	87.5
Wang et al. (65)	China	Cohort	Real-time measurement	PM_10_, PM_2.5_, SO_2_, NO_2_, CO, O_3_	PTB	Generalized additive model	2018–2019	424	17	4	87.5
Xiao et al. (66)	China	Birth records	Satellite-derived estimates or central-site measurements	PM_2.5_	PTB, LBW	Linear and logistic Regressions	2011–2014	132,783	7,117	5.36	87.5
Zang et al. (67)	China	Cohort	Daily measurement	PM_2.5_, PM_10_, SO_2_, NO_2_, CO, O_3_	SB	Logistic regression	2015–2017	59,868	587	0.98	75
Arroyo et al. (68)	Spain	Time-series	Daily measurement	PM_2.5_, NO_2_, O_3_	PTB, LBW	Poisson regression models	2001–2009	298,705	64,169	21.5	62.5
DeFranco et al. (69)	United States	Cohort	Monitoring stations	PM_2.5_	SB	Generalized estimating equation	2005–2010	349,188	1,848	0.53	87.5
Coker et al. (70)	United States	Cohort	Land use regression	PM_2.5,_ NO_2_, NO	LBW	Bayesian profile regression	2000–2006	804,726	16,694	2.07	62.5
Yuan et al. (71)	China	Cohort	Satellite-based estimates and ground-level measurements	PM_2.5_	PTB, LBW	multiple linear models	2013–2016	3,692	274	7.4	87.5
Li et al. (72)	China	Time-series	Weekly air quality data	PM_2.5_, PM_10_, O_3_, SO_2_, NO_2, CO_	PTB	Distributed lag non-linear model	2016–2019	120,446	5,408	4.5	87.5
Rammah et al. (73)	United States	Cohort	Daily measurement	O_3_	SB	Multipollutant models and measure modification	2008–2013	358,366	1,599	0.45	87.5
Nahian et al. (28)	Bangladesh	Birth records	Air quality index	PM_2.5_, PM_10_, O_3_, SO_2_, NO_2_	PTB, LBW	Logistic regression model	2014–2017	3,206	1,287	40.1	87.5
Mitku et al. (74)	South Africa	Cohort	Land use regression	PM_2.5,_ SO_2_	PTB, LBW	Generalized Structure Equation	2013–2017	996	206	20.7	62.5
Li et al. (75)	China	Cohort	Satellite remote sensing, meteorological and land use information	PM_2.5_, PM_10_	PTB	Cox proportional hazard regression	2013–2014	1,240,978	100,433	8.1	87.5
Bachwenkizi et al. (76)	Africa	Cross-sectional	Global exposure assessment	PM_2.5_, O_3_	PTB, LBW	Multivariable logistic regression	2005–2015	131,594	17,591	13.4	87.5
Chu et al. (77)	China	Cohort	Satellite remote sensing	PM_2.5_	PTB	Cox proportional hazard models	2009–2011	5,976	443	7.4	87.5
Han et al. (78)	China	Cohort	Inverse distance weighting	PM_10_, O_3_	PTB	Logistic and linear regression models	2014–2016	6,693	638	9.53	87.5
Zhang et al. (79)	China	Birth records	Daily measurement	O3	PTB, LBW	Cox proportional hazard models	2016–2019	34,122	2,829	8.3	75
Kim et al. (80)	Korea	Birth records	Daily measurement	PM_10_	PTB, LBW	Linear and logistic regression	2010–2013	1,742,183	148,086	8.5	62.5
Liang et al. (81)	China	Cohort	Air monitoring stations	PM_2.5_	PTB, LBW	Cox proportional hazards regressions	2014–2017	1,455,026	121, 646	8.4	62.5
Chen et al. (82)	China	Cohort	Daily measurement	PM_2.5_, PM_10_, SO_2_, CO, O_3,_ NO_2_	PTB, LBW	Cox proportional hazards regression	2014–2016	10,960	291	2.7	87.5
Johnson et al. (83)	United States	Birth records	Air survey and regulatory monitors	PM_2.5,_ NO_2_	PTB	Logistic mixed models	2008–2010	132,654	10, 271	7.7	87.5
Siddika et al. (84)	Finland	Cohort	Regional-to-city-scale dispersion modelling and land-use regression	PM_2.5_, PM_10_, NO_2_	PTB	Dispersion modelling and land-use regression	1984–1990	2,568	195	7.6	75
Sun et al. (85)	China	Cohort	Real-time measurement	PM_2.5_, PM_10_, O_3_, SO_2_, NO_2_	PTB	logistic regressions model	2013–2017	6,275	372	5.9	75
Hao et al. (86)	United States	Cohort	Ensemble-based models	NO_2_, PM_2.5,_ O_3_	PTB	Logistic regression model	2000–2015	596,926	41,936	7.03	62.5
Fang et al. (87)	China	Longitudinal population study	Daily measurement	PM_2.5_	PTB, LBW	Generalized additive distributed lag models	2014–2016	10,738	303	2.8	75

NB: PTB, Preterm birth; LBW, Low birth weight; SB, Stillbirth; −, not reported.

### 2.6 Data extraction and management

Data extraction was conducted by two authors (BD and AKG) using a data extraction tool. Any disagreements between the two data extractors were resolved through consensus or with the involvement of a third author (GB). The following information was extracted from the selected studies: author information, study setting, study country, type of study, pollutants, outcomes, statistical methods, study periods, sample size, cases, prevalence, and quality scores. The extracted data was organized in a table format (Table 1).

### 2.7 Statistical methods and data analysis

The extracted data from Microsoft Excel was transported to STATA version 17 for analysis. The Index of heterogeneity (*I*^2^ statistics) was used to assess variations among the included studies, where values of 25–50%, 50–75%, and >75% indicated low, moderate, and high heterogeneity, respectively (27). The metaprop command in STATA was used to estimate the pooled prevalence. Subgroup analysis were conducted to explore potential variations in the pooled prevalence based on study countries and the nature of the outcomes. Sensitivity analysis was performed to assess the effect of each individual study on the estimated pooled results. To evaluate publication bias, a funnel plot test and Egger’s regression test were used. A meta-regression was employed to identify potential sources of heterogeneity. Finally, the findings of this study are presented using tables, figures, forest plots, and descriptive texts.

## 3 Results

### 3.1 Overview of search process

We identified a total of 79,356 studies using a database and through direct Google and citation searching. After duplicate records were removed, 51,592 records were screened for this review. According to the records, only 32,947 studies were sought for retrieval. After being identified for retrieval, 15,063 studies were evaluated for eligibility. Following eligibility, a total of 15,021 studies were excluded due to differences in outcome interest and population differences. Ultimately, a total of 42 studies were included in this review from database sources. In addition to the database sources, seven studies were included in this review from direct Google and citation searching. Finally, a total of 49 articles were included in this study, as presented in the PRISMA flowchart (Figure 1).

### 3.2 Characteristics of the eligible studies

The majority of the included studies were conducted using birth cohort studies. This meta-analysis included a total of 21,019,317 study participants. The majority of the studies were conducted in China (*n* = 26) and the United States (*n* = 10). This meta-analysis examined the association between ambient air pollutants (PM_2.5_, PM_10_, SO_2_, NO_2,_ O_3_, CO) and adverse birth outcomes (preterm birth, low birth weight, and stillbirths). In this study, the main exposure assessment methods were the daily mean concentration of air pollutants, monitoring stations, land use regression model, and real-time measurement. Among the included studies, Bangladesh had the highest at least one birth outcome (40%) (28), while the United States had the lowest rate (0.44%) (29). The quality score of the included studies was between the ranges of 62.5 and 87.5% (Table 1).

### 3.3 Meta-analysis

**Figure 2:**
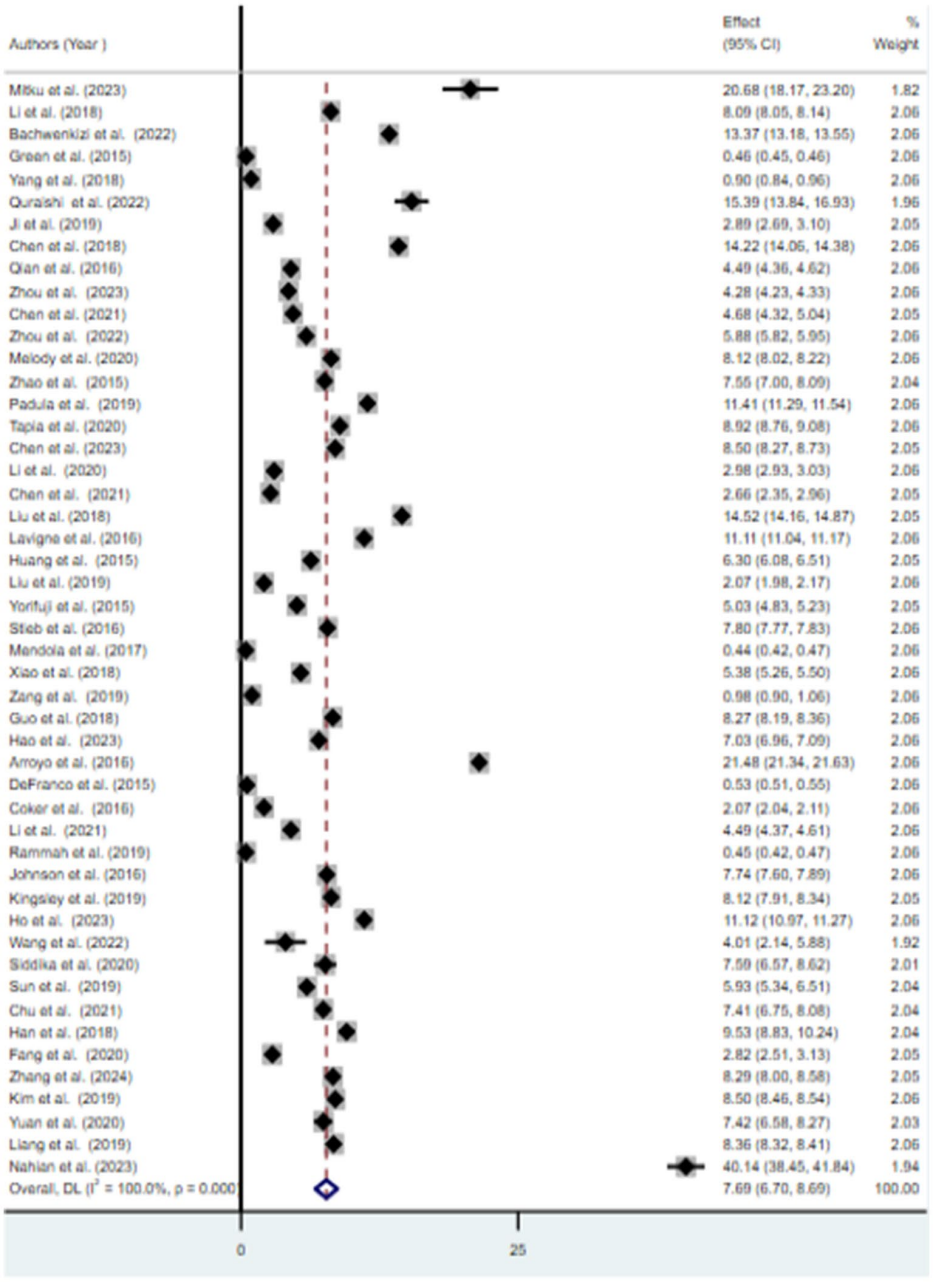
Forest plot of the overall prevalence for at least one adverse birth outcome, 2024.

**Figure 3:**
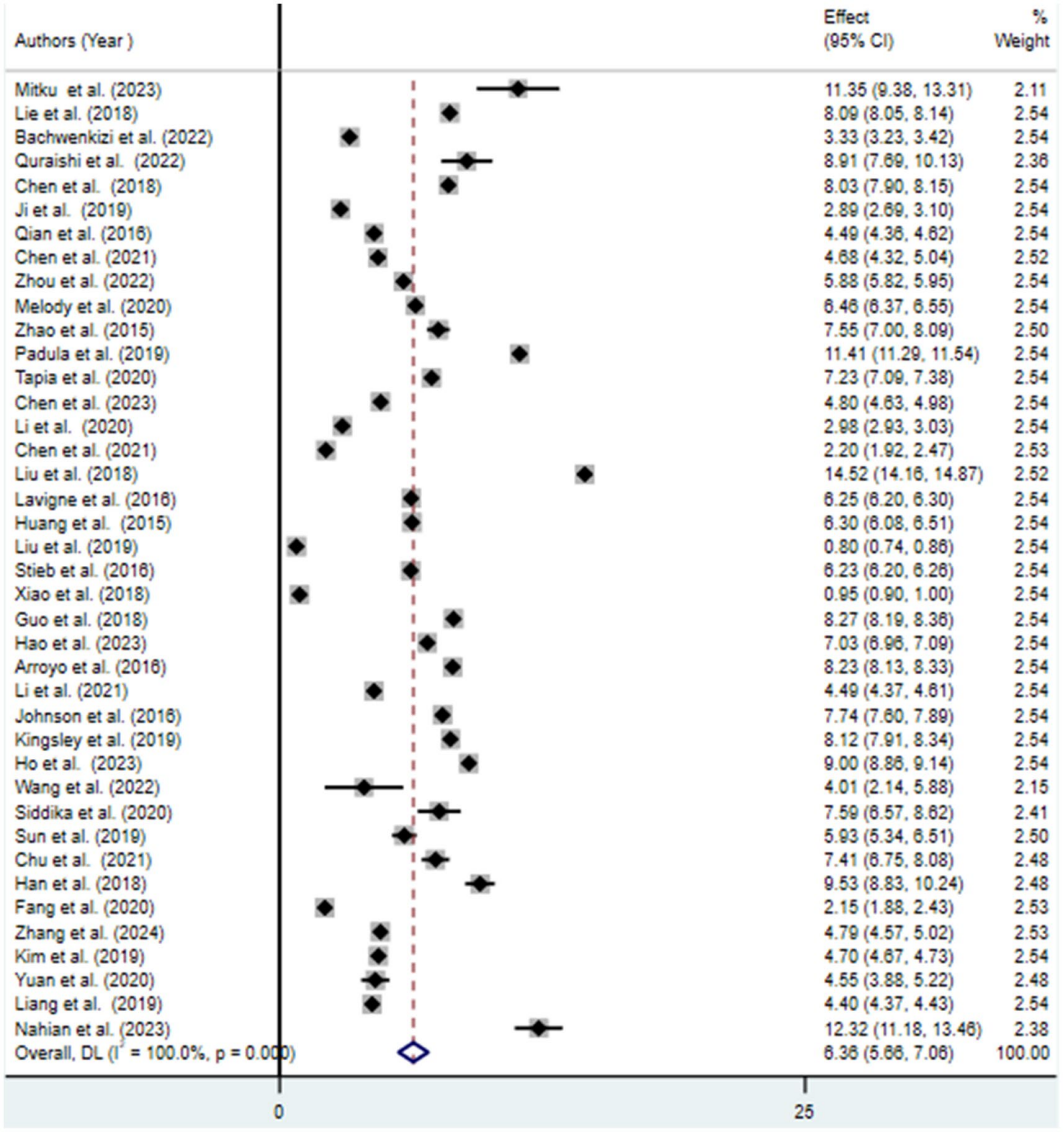
Forest plot for the association between ambient air pollution and preterm birth, 2024.

**Figure 4:**
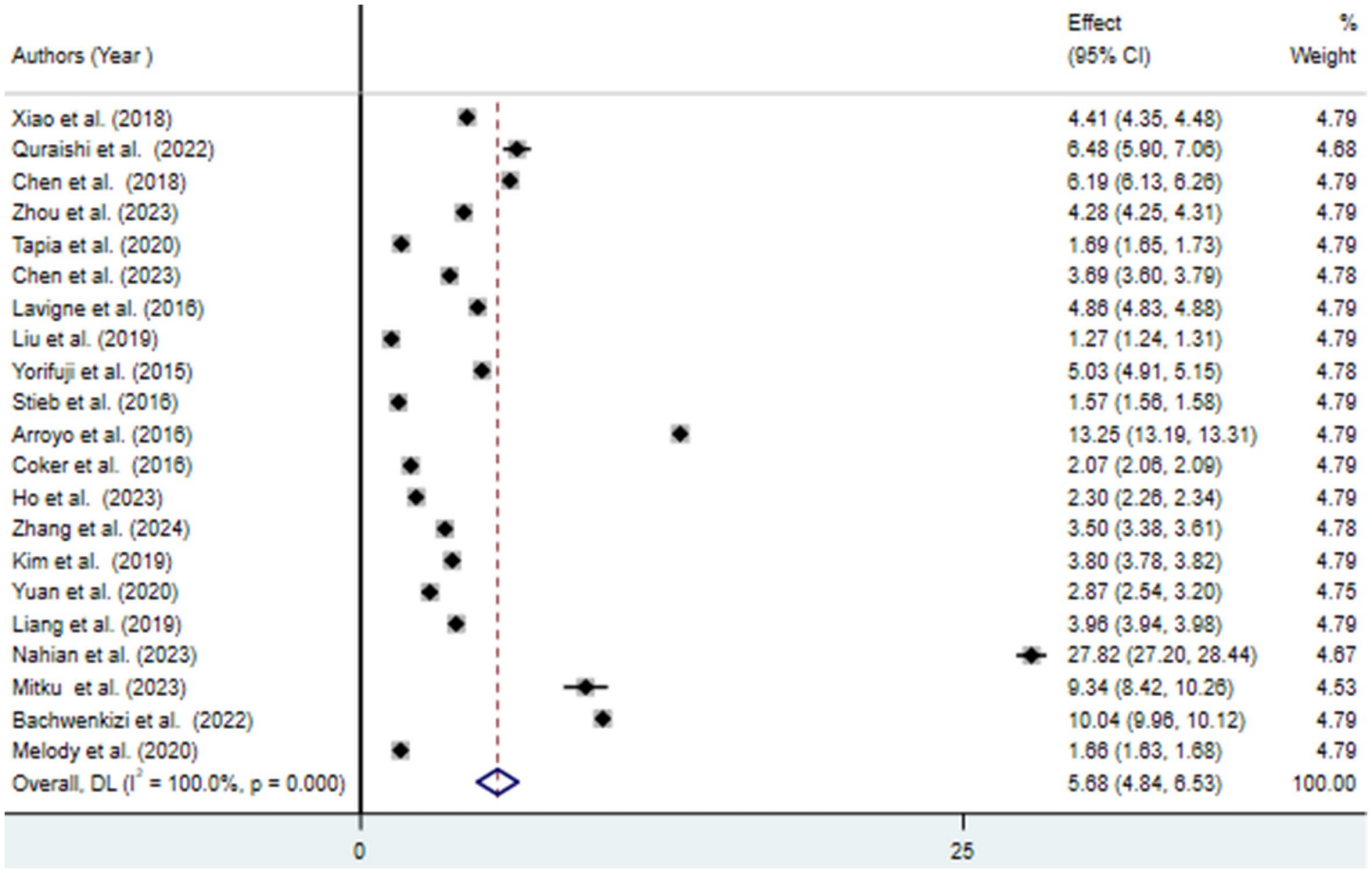
Forest plot for the association between ambient air pollution and low birth weight, 2024.

**Figure 5:**
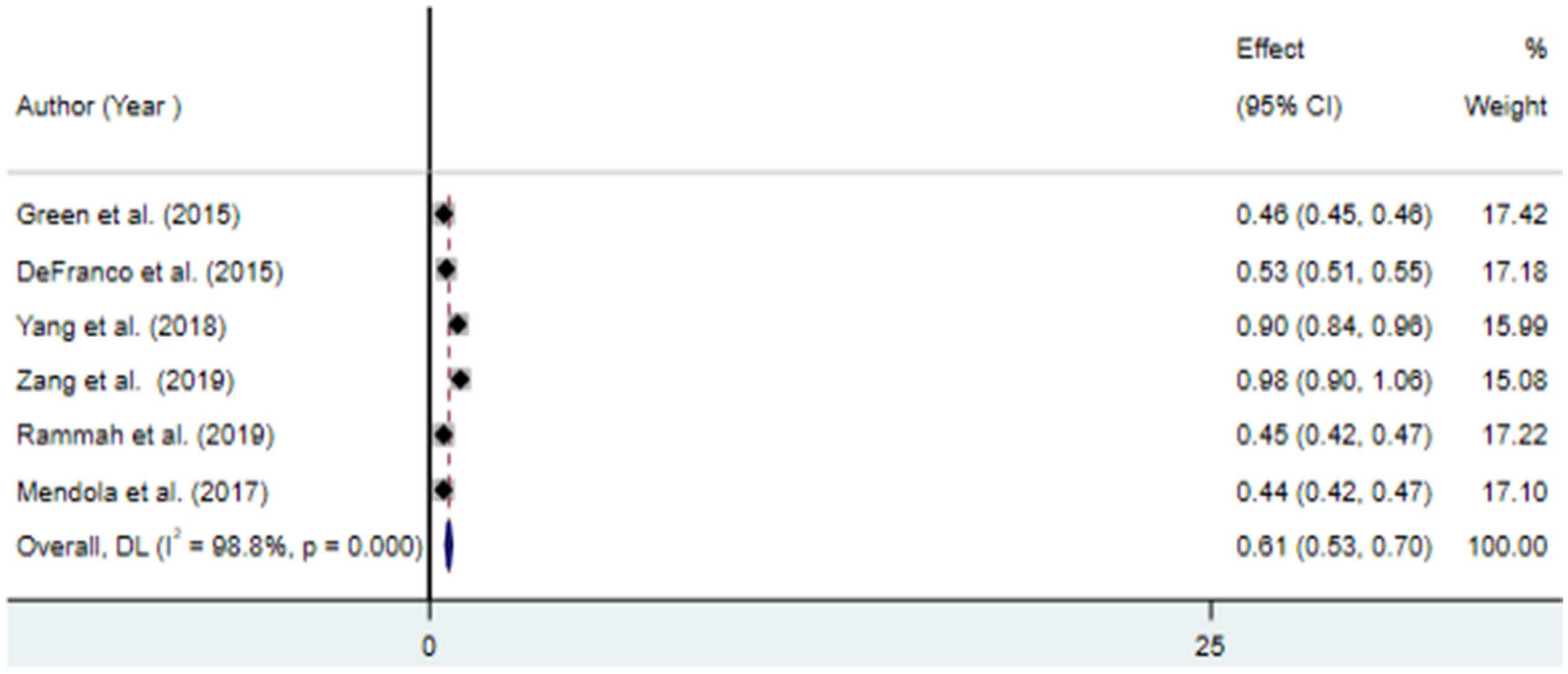
Forest plot for the association between ambient air pollution and stillbirth, 2024.

The findings from the random effects model indicated that the pooled prevalence of at least one adverse birth outcome was 7.69% (95% CI: 6.70–8.69), with high heterogeneity (*I*^2^ = 100%, *p-value* < 0.001) (Figure 2). In this meta-analysis, high pooled prevalence was found in preterm birth (6.36%) (Figure 3), followed by low birth weights (5.07%) (Figure 4) and stillbirth (0.61%) (Figure 5). Subgroup analysis based on study country: the highest pooled prevalence of at least one adverse birth outcome was observed in Bangladesh at 40.14% (95% CI: 38.45–41.84) and in Spain at 21.5% (95% CI: 21.34–21.63). In contrast, the lowest pooled prevalence of at least one adverse birth outcome was observed in Japan at 5.03% (95% CI: 4.83–5.24) and the United States at 5.12% (95% CI: 4.22–6.02). In addition, subgroup analysis based on the nature of outcomes found that preterm birth and low birth weight were at 11.1% (95% CI: 9.85–12.35), preterm birth at 6.96% (95% CI: 5.86–8.66), low birth weight at 3.79% (95% CI: 2.0–5.59), and stillbirth at 0.615% (95% CI: 0.53–0.699) (Table 2).

**Table 2:** Subgroup analysis of the association between ambient air pollution and at least one adverse birth outcome, 2024.

Variable	Number of studies	OR (95%CI)	Heterogeneity	
			*I* ^2^	*p-value*
Region/country				
Africa	2	16.9 (9.5–24.1)	96.9	<0.001
China	26	5.72 (4.6–6.9)	100	<0.001
United States	10	5.12 (4.22–6.02)	100	<0.001
Australia	2	11.2 (5.2–17.15)	100	<0.001
Peru	1	8.92 (8.8–9.08)	–	–
Canada	2	9.45 (6.22–12.69)	100	<0.001
Japan	1	5.03 (4.83–5.24)	–	–
Spain	1	21.5 (21.24–21.63)	–	–
Vietnam	1	11.12 (10.97–11.27)	–	–
Finland	1	7.59 (6.57–8.62)	–	–
Korea	1	8.5 (8.46–8.54)	–	–
Bangladesh	1	40.14 (38.45–41.84)	–	–
Outcome				
PTB and LBW	20	11.1 (9.85–12.35)	100	<0.001
PTB	20	6.96 (5.86–8.66)	99.9	<0.001
LBW	3	3.79 (2.0–5.59)	100	<0.001
SB	6	0.615 (0.53–0.699)	98.8	<0.001

NB: PTB, Preterm birth; LBW, Low birth weight; SB, Stillbirth.

In this study, a meta-regression analysis was conducted using the study country and nature of outcomes as factors to identify the source of heterogeneity. The finding revealed that the study country was not a statistically significant source of heterogeneity (*p* = 0.196), but the outcome nature was found to be a statistically significant source of heterogeneity (*p* < 0.001).

A sensitivity analysis was also performed to evaluate a single study effect on the overall results. The analysis showed for the overall prevalence of at least one adverse birth outcome a slightly broader confidence interval of 7.69% (95% CI: 6.05–8.98) compared to the original pooled prevalence of 7.69% (95% CI: 6.70–8.69), but it does not suggest strong evidence for single study effects. Similarly, sensitivity analysis was also conducted for preterm birth, low birth weight, and stillbirth to examine the effect of a single study on the overall prevalence. The findings suggested that there is no evidence for a single study effect on the overall pooled prevalence (Supplementary material 1).

**Figure 6:**
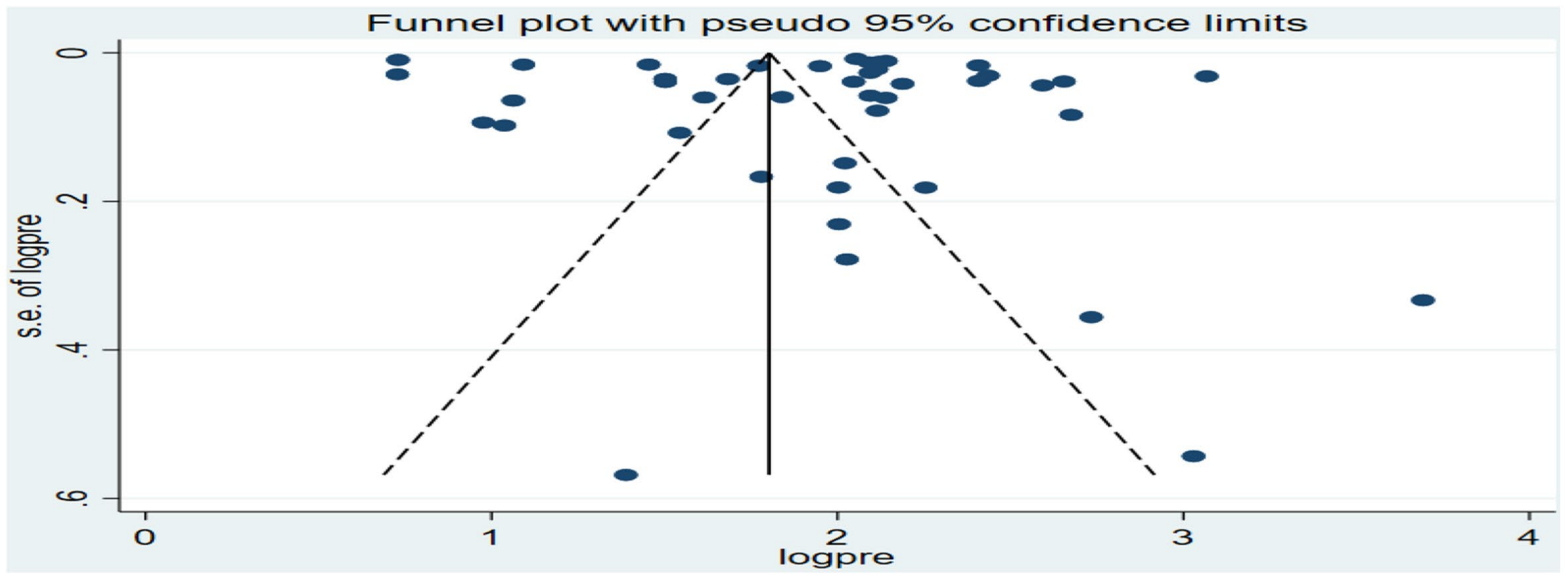
Funnel plot for the association between ambient air pollution and at least one adverse birth outcome, 2024.

The funnel plot for the overall analysis showed an asymmetrical distribution as visualized of the included articles, revealing the potential of publication biases (Figure 6). However, the Egger-regression test confirmed that there was no statistically significant presence of publication bias (*p*-value = 0.1001).

**Figure 7:**
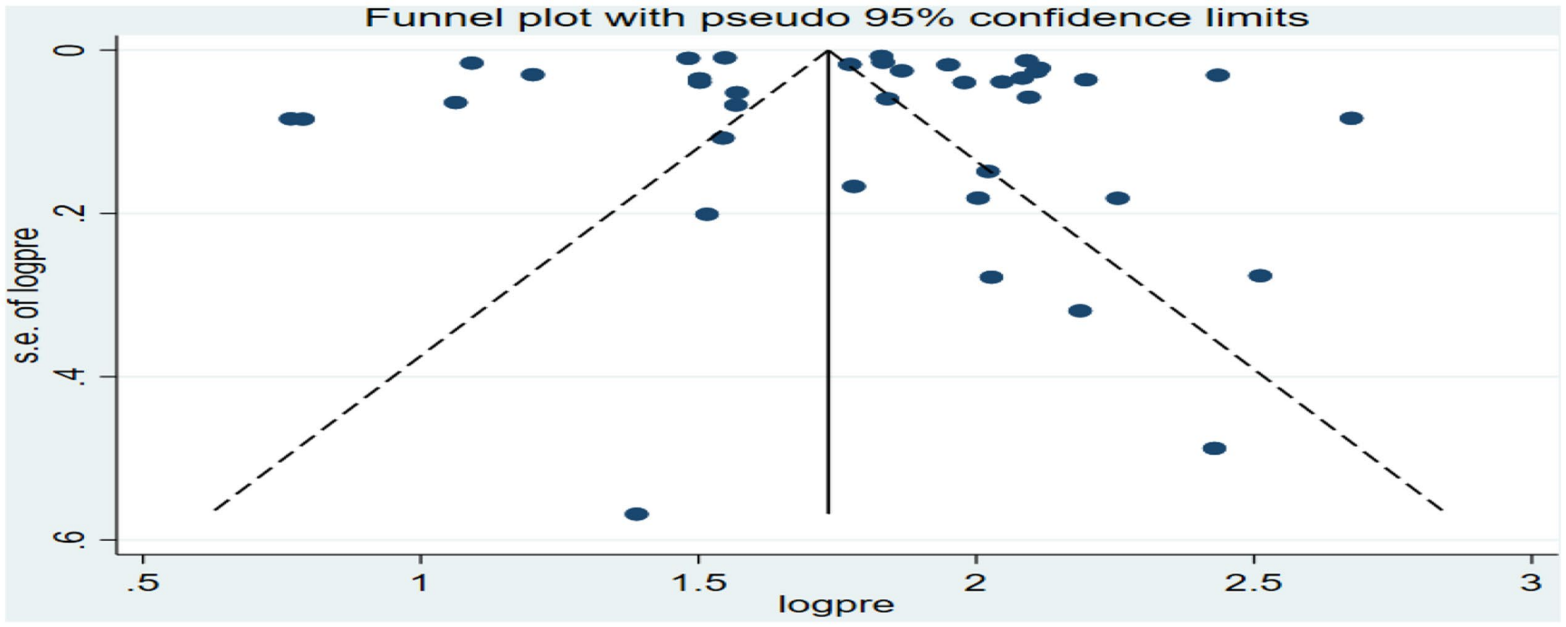
Funnel plot of the association between ambient air pollution and preterm birth, 2024.

Similarly, funnel plots and Egger-regression tests were conducted to assess publication biases for the specific birth outcomes of preterm birth and low birth weights. For preterm birth, the funnel plots showed an asymmetrical distribution as visualized of the included articles (Figure 7), but the Egger-regression test confirmed that there was no statistically significant (*p*-value = 0.2087) for the presence of publication bias.

**Figure 8:**
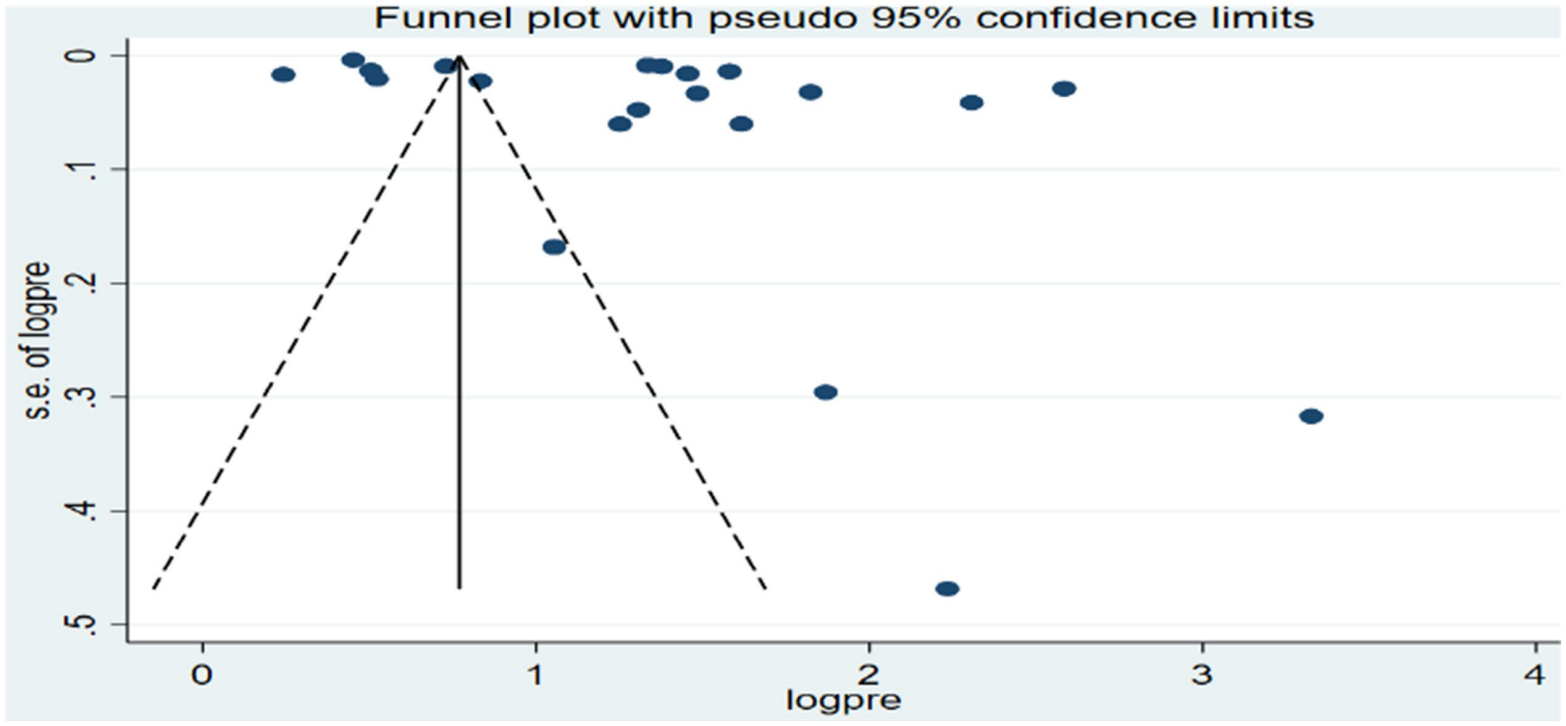
Funnel plot of the association between ambient air pollution and low birth weight, 2024.

In contrast, for low birth weights, the funnel plot also showed an asymmetrical distribution as visualized of the included articles (Figure 8), and the Egger-regression test confirmed the presence of publication bias with statistical significance (*p*-value = 0.0017). To address the publication bias identified for the low birth weight outcome, Duval and Tweedie’s “trim and fill” method was conducted (Supplementary material 2).

### 3.4 Factors associated with adverse birth outcomes

**Figure 9:**
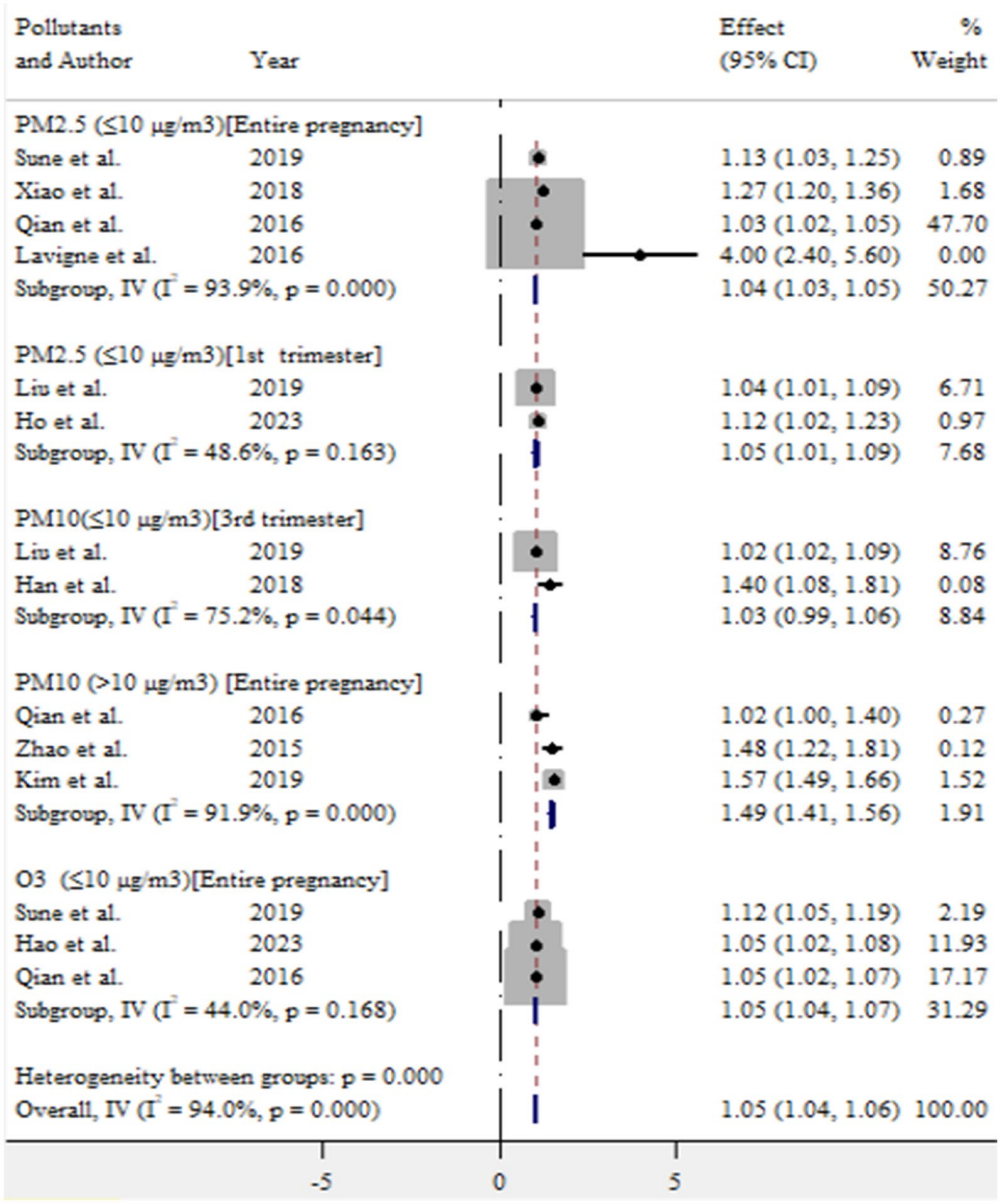
Pooled effect size of ambient air pollution associated with preterm birth, 2024.

In this meta-analysis, exposure to ambient air pollution, such as PM_2.5_, PM_10_, and O_3_, was statistically significant for adverse birth outcomes (preterm birth and low birth weight). For preterm birth, exposure to PM_2.5_ (≤10 μg/m^3^) during entire pregnancy, PM_2.5_ (≤10 μg/m^3^) in first trimester, PM_10_ (>10 μg/m^3^) during entire pregnancy, and O_3_ (≤10 μg/m^3^) during entire pregnancy increased the risk by 4% (OR = 1.04, 95% CI: 1.03–1.05), 5% (OR = 1.05, 95% CI: 1.01–1.09), 49% (OR = 1.49, 95% CI: 1.41–1.56), and 5% (OR = 1.05, 95% CI: 1.04–1.07), respectively (Figure 9).

**Figure 10:**
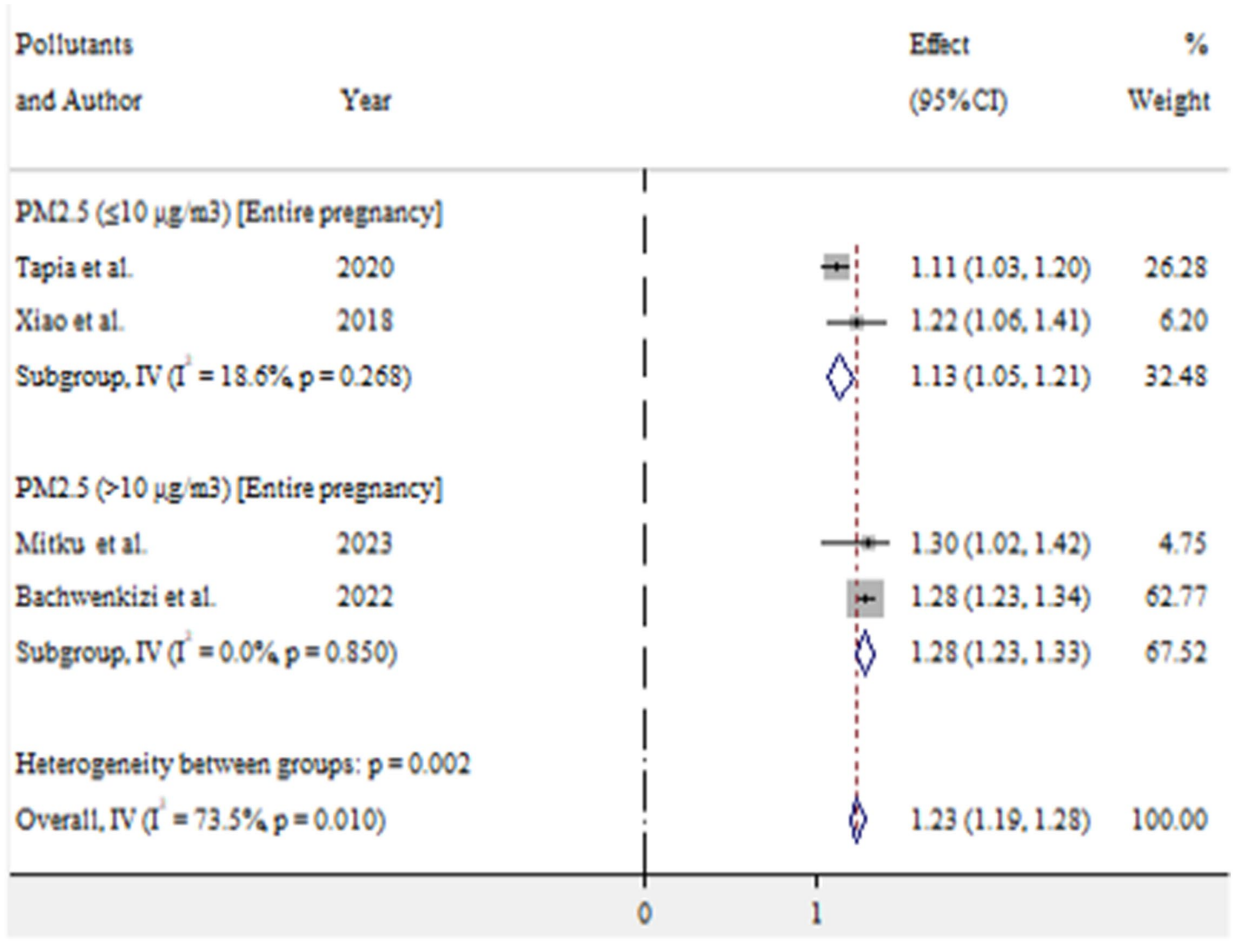
Pooled effect size of ambient air pollution associated with low birth weight, 2024.

For low birth weight, exposure to PM_2.5_ (≤10 μg/m^3^) and PM_2.5_ (>10 μg/m^3^) during entire pregnancy was found to increase the risk by 13% (OR = 1.13, 95% CI 1.05–1.21) and 28% (OR = 1.28, 95% CI 1.23–1.33), respectively (Figure 10).

## 4 Discussion

This systematic review and meta-analysis aimed to estimate the pooled association between exposure to ambient air pollution and adverse birth outcomes (preterm birth, low birth weight, and stillbirth), as well as to identify predictive factors. The pooled prevalence of at least one adverse birth outcome was found to be 7.69% (95% CI: 6.70–8.69), with notable extreme heterogeneity among the included studies (*I*^2^ = 100, *p* < 0.001). Specifically, exposure to ambient air pollution was associated with a 6.36% (95% CI: 5.66–7.06) increased risk of preterm birth, a 5.07% (95% CI: 4.32–5.81) increase in low birth weight, and a 0.65% (95% CI: 0.53–0.7) increase in the risk of stillbirth. These findings suggest that exposure to ambient air pollution during pregnancy negatively affects various birth outcomes (30, 31).

Daba et al. (32) support the present findings, reporting a 15.5% (95% CI: 12.6–18.5) prevalence of adverse pregnancy outcomes linked to indoor air pollution exposure. The WHO reported low birth weight at 15.5% globally, 16.5% in developing countries, and 7% in developed countries in 2015. The current findings are also supported by numerous studies indicating that exposure to ambient air pollution increases the risk of preterm birth, low birth weight, and stillbirth (30, 31, 33). Variations in findings may be attributed to different factors such as maternal educational level, age differences, socioeconomic conditions, type of pollutants, and the duration and level of exposure during the perinatal period (34, 35). Dzekem et al. (36) highlighted the need to address disparities, such as socioeconomic issues, when examining the relationship between air pollution exposure and pregnancy outcomes. Therefore, it is crucial to ensure that the interaction between pregnant women and their environment is safe and well-maintained to prevent adverse effects.

In this study, the heterogeneity among the included studies was significantly high, a finding that is supported by previous research (14, 31–33, 37, 38). This variability may be attributed to various factors, including differences in study settings, designs, and exposure assessment methods. To identify the potential sources of this heterogeneity, we conducted a subgroup analysis based on the study country and the type of adverse birth outcomes. Subsequently, the meta-regression analysis confirmed that the primary source of heterogeneity was related to the nature of the adverse birth outcomes. This may be linked to the levels and types of pollutants, as well as the conditions of pregnant women.

In this meta-analysis, exposure to PM_2.5_ (≤10 μg/m^3^) throughout the entire pregnancy and during the first trimester was associated with an increased risk of preterm birth, with an OR of 1.04 (95% CI: 1.03–1.05) and 1.05 (95% CI: 1.01–1.04), respectively. Sapkota et al. (37) reported that exposure to PM_2.5_ at levels of 10 μg/m^3^ during pregnancy increased the risk of preterm birth with an OR of 1.15 (95% CI: 1.14–1.16). Liu et al. (39) found a similar positive association, reporting an OR of 1.15 (95% CI: 1.07–1.23) for PM_2.5_ exposure during pregnancy. Additionally, Lamichhane et al. (3) estimated an OR of 1.14 (95% CI = 1.06–1.22) for preterm birth per 10 μg/m^3^ increase in PM_2.5_ exposure during the entire pregnancy. Therefore, these findings highlighted the importance of protecting pregnant mothers from PM_2.5_ exposure to reduce the risk of adverse outcomes of preterm birth (39–41).

In this study, we found a 49% increase in the risk of preterm birth for each >10 μg/m^3^ increase in PM_10_ exposure during the entire pregnancy. Stieb et al. (1) reported a high risk of preterm birth associated with a 20 μg/m^3^ increase in PM_10_ over the same period. Similarly, Lamichhane et al. (3) noted that exposure to PM_10_ increased the risk of preterm birth by 23% for each 10 μg/m^3^ increment. The slight difference in findings may be attributed to variation in exposure levels and duration. Therefore, addressing ambient air pollution is essential for reducing the occurrence of preterm birth.

In the present study, exposure of O_3_ at levels of ≤10 μg/m^3^ during pregnancy was positively associated with a 5% increased risk of preterm birth, with (OR = 1.05, 95% CI: 1.04–1.07). This finding is consistent with previous research (31, 33), which suggests that exposure to ozone throughout pregnancy may significantly elevate the risk of preterm birth. These findings underscoring the need for protective measures to minimize pregnant women’s exposure to ozone.

In this meta-analysis, maternal exposure to PM_2.5_ during the entire pregnancy at levels of >10 μg/m^3^ was associated with a 28% increase in the risk of low birth weight (OR = 1.28, 95% CI: 1.23–1.33). Additionally, exposure to PM_2.5_ (≤10 μg/m^3^) also showed a 13% increase in risk (OR = 1.13, 95% CI: 1.05–1.21). These findings suggest that as PM_2.5_ exposure increases, the risk of low birth weight also increases. Supporting this, Zhu et al. (4) reported a 5% increase in low birth weight per 10 μg/m^3^ increment in PM_2.5_ exposure during the entire pregnancy (OR = 1.05, 95% CI: 1.02–1.07). The difference in findings may be attributed to variations in exposure assessment methods. Thus, exposure to PM_2.5_ throughout pregnancy could significantly impact the final birth weight.

### 4.1 Limitation of the study

This study focusses exclusively on studies conducted in the English language. Additionally, it does not explore the underlying mechanisms that link ambient air pollution to adverse birth outcomes. Furthermore, this study also focused on selected adverse birth outcomes, but other birth outcomes like congenital anomalies or birth defects and others might be important.

## 5 Conclusion

This meta-analysis highlighted a significant association between ambient air pollution and adverse birth outcomes. In this study, PM_2.5_, PM_10_, and O_3_ were found to be positively associated with these adverse birth outcomes (preterm birth and low birth weight). Given these findings, it is essential for healthcare professionals, the Ministries of Health, non-governmental organizations, and other relevant stakeholders to implement compressive public health interventions aimed at reducing the incidence of adverse birth outcomes related to ambient air pollution. Such interventions could include policies to improve air quality, strict regulations, public awareness campaigns, and targeted support for vulnerable populations of pregnant women. Furthermore, to gain a deeper understanding of the mechanisms underlying the association between ambient air pollution and adverse birth outcomes, future research is highly recommended. Investigating these mechanisms will provide valuable insights that can help inform more effective strategies for mitigating the risks associated with air pollution during pregnancy. In addition, future researchers are encouraged to investigate the impact of ambient air pollution on congenital anomalies and other significant adverse birth outcomes.

## Data Availability

The original contributions presented in the study are included in the article/Supplementary material, further inquiries can be directed to the corresponding author.
