# Supplementary material for: Exposure to ambient air pollutions and its association with adverse birth outcomes: a systematic review and meta-analysis of epidemiological studies
Source: Front Public Health. 2024 Nov 13;12:1488028. doi: 10.3389/fpubh.2024.1488028 (PMC11600733; doi:10.3389/fpubh.2024.1488028)
Supplement: Supplementary file 1 [file Data_Sheet_1.docx]

Supplementary File: Sensitivity analysis for association between ambient air pollution and at least one birth outcomes, 2024.

Supplementary File: Sensitivity analysis for association between ambient air pollution and preterm birth, 2024.

Supplementary File: Sensitivity analysis for association between ambient air pollution and low birth weight, 2024.

Supplementary File: Sensitivity analysis for association between ambient air pollution still birth, 2024.
